# Sexuality in the Muslim community during Ramadan

**DOI:** 10.1192/j.eurpsy.2022.2069

**Published:** 2022-09-01

**Authors:** N. Sayari, A. Maamri, A. Hajri, R. Haoua, H. Zalila

**Affiliations:** Razi Hospital, Emergency And Outpatient Departement, manouba, Tunisia

**Keywords:** Ramadan, sexual behaviour, fasting, sexuality

## Abstract

**Introduction:**

Ramadan is a holly month for Muslims. Able-bodied adults fast and abstain from sexual activities from dawn to sunset. These hasty lifestyle changes have a major impact on the sexual life of the Muslim community.

**Objectives:**

To assess the impact of Ramadan on sexual activity in a Tunisian community.

**Methods:**

A cross-sectional study was conducted among married Muslim volunteers in Tunisia. The data was collected with an anonymous self-completed questionnaire, one week before Ramadan (W-1) and the fourth week of Ramadan (W4).

**Results:**

We included 100 person in this study. The sample consisted of 59 females and 41 males. The average age was 40.3 years. During Ramadan, sexual intercourse happened more often on weekends (p=0.009) and mostly during the second part of the night (p ˂0.001). Data suggest a disturbed sex life with less satisfaction about sexual life compared to W-1 (p<0.05). Monthly sexual intercourse frequency dropped from 5.76 coitus/month on W-1 to 3.27 on W 4 (p<0.05) and Duration of sex from 9.45 minutes to 6.85 minutes (p˂0.001). Couples communicated less about sex (p=0.004). Sexual abstinence was more frequent (p=0.016). Preliminaries were less performed (P=0.01) and were shorter (p˂0.001).Oral sex was less frequent as well as some sexual positions: fellatio (p=0.01) and cunnilingus (p=0.002), sexual positions of Andromache (P=0.002), posterior vaginal (P˂0.001) and lateral (P=0.001). The participants used less pornography (p=0.007).

**Conclusions:**

This study demonstrated the deleterious impact of the lifestyles changes in Ramadan on the sexual life. Better sexual and religious education is recommended to prevent sexual dysfunctions.

**Disclosure:**

No significant relationships.

